# Cytocentric measurement for regenerative medicine

**DOI:** 10.3389/fmedt.2023.1154653

**Published:** 2023-04-27

**Authors:** Alicia D. Henn, Taci Pereira, Joshua Hunsberger, Kunal Mitra, Zohreh Izadifar, Sita Somara, Lisa Lindström, Thomas Forest Farb-Horch, Jake Boy, George F. Muschler, Steven R. Bauer, Randy Yerden

**Affiliations:** ^1^ BioSpherix, Ltd. Parish, Parish, NY, United States; ^2^ 3DSystems, Rock Hill, SC, United States; ^3^ RegenMed Development Organization, Winston-Salem, NC, United States; ^4^ Biomedical Engineering, Florida Institute of Technology, Melbourne, FL, United States; ^5^ Wyss Institute, Harvard University, Boston, MA, United States; ^6^ Vigene Biosciences, Rockville, MD, United States; ^7^ Phase Holographic Imaging, Lund, Sweden; ^8^ Thrive Bioscience, Inc. Beverly, MA, United States; ^9^ Scientific Bioprocessing, Pittsburgh, PA, United States; ^10^ Cell X Technologies, Cleveland, OH, United States; ^11^ Wake Forest Institute for Regenerative Medicine, Winston-Salem, NC, United States

**Keywords:** regenerative medicine, tissue engineering, cell culture techniques, organoids, stem cells, Sensors

## Abstract

Any Regenerative Medicine (RM) business requires reliably predictable cell and tissue products. Regulatory agencies expect control and documentation. However, laboratory tissue production is currently not predictable or well-controlled. Before conditions can be controlled to meet the needs of cells and tissues in culture for RM, we have to know what those needs are and be able to quantify them. Therefore, identification and measurement of critical cell quality attributes at a cellular or pericellular level is essential to generating reproducible cell and tissue products. Here, we identify some of the critical cell and process parameters for cell and tissue products as well as technologies available for sensing them. We also discuss available and needed technologies for monitoring both 2D and 3D cultures to manufacture reliable cell and tissue products for clinical and non-clinical use. As any industry matures, it improves and standardizes the quality of its products. Cytocentric measurement of cell and tissue quality attributes are needed for RM.

## Sensing the needs of cells in culture

We previously published the cytocentric principles for regeneration of cell and tissue products (1) which outlines the basic needs of cells in culture. Cells need protection from contamination, physiologic simulation, and full-time conditions for cultures that are optimal, individualized, and dynamic. Growing cells into tissues requires sensing those needs, then meeting them at all times during production.

In forming a cultured tissue, cells have a tough job. They must overcome all the stresses of a violent primary isolation process to expand in number, connect with other cells, heal, and function as a tissue again. It is our challenge to provide them all they need to recover, grow, and form the most reliable and functional tissue product.

**Figure 1:**
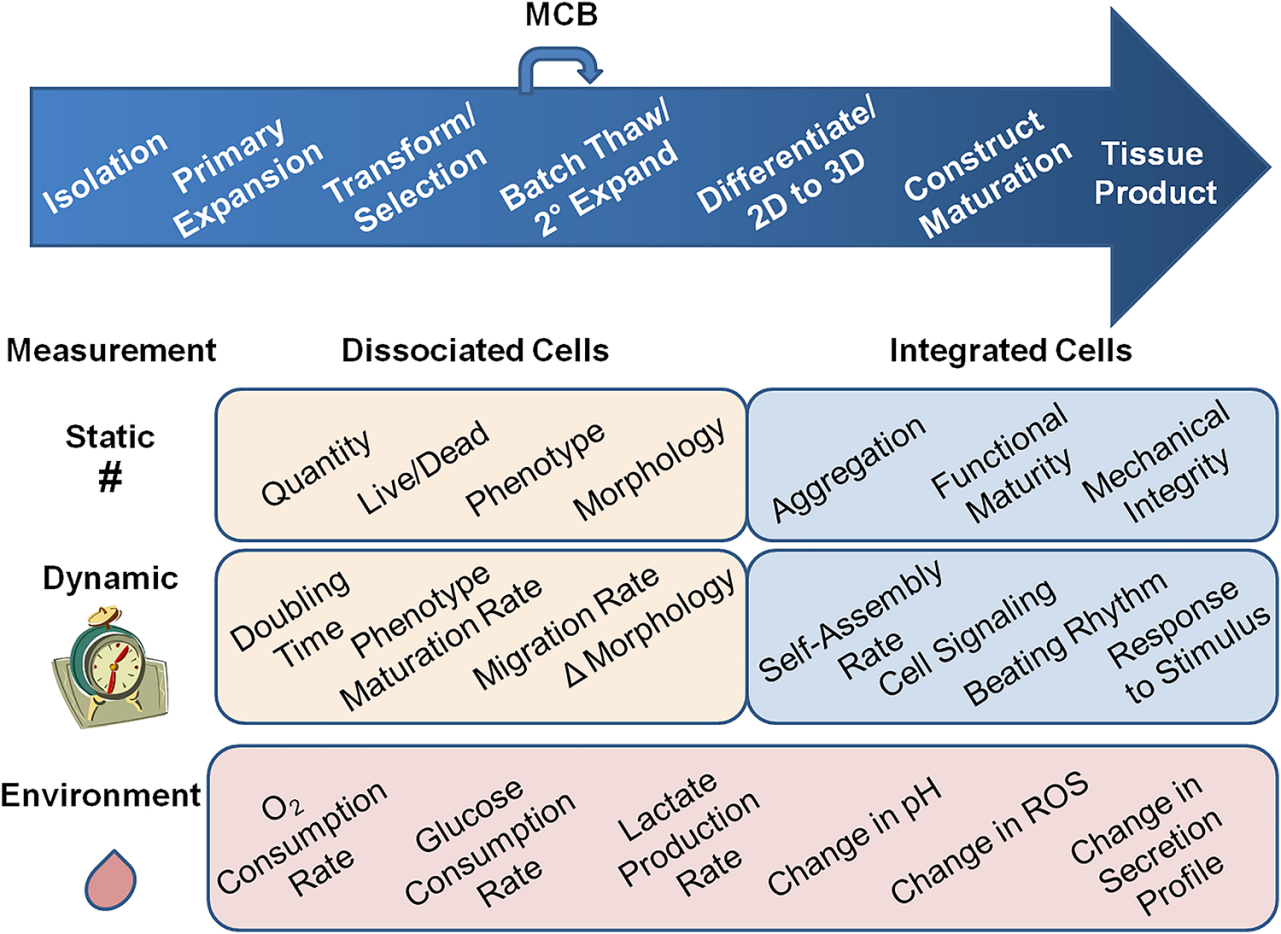
Types of measurements for regenerative medicine tissue production quality attributes. Processes for RM tissue production are as diverse as the tissues they produce; however many of them have these general steps. Cells are isolated, expanded *in vitro*, and some processes have manipulation steps, i.e. generation of iPSC and re-differentiation. There there is a selection step and cells may go into a Master Cell Bank (MCB) for storage, whether the process is allogeneic or patient-specific. For producing the organ, the cells may undergo a secondary expansion before being loaded into a scaffold to make a construct and placed in a bioreactor. There may be more construct maturation steps as well as construct folding or assembly into higher-order structures. At each step away from simple 2D culture, measurement of the quality attributes, and the needs of cells, gets more difficult. There are static measures like cell number, live/dead, phenotype, and morphology that are well established for 2D cultures, but need more development for higher order structures. In addition, there are static quality attributes like cell aggregation, appearance of functional maturity, and mechanical integrity that could be applied to cells integrated into 3D and higher-order structures. Dynamic measurements add the element of time. Static measurements in 2D cultures become doubling time, maturation rates, migration rates, and change in morphology. Dynamic measures may better predict later product QAs. In addition to these attributes, cells integrated into constructs may have self-assembly rates, cell signaling changes, and functional changes like acquiring a heartbeat, and responses to stimulus that reflect proper whole-organ function. Each of these attributes need to be measured as a reflection of cell quality. Environmental QAs like oxygen and glucose consumption rate, lactate production rate, change in pH, and reactive oxygen species (ROS), can be critical process parameters (CPPs). Also production of soluble factors that indicate organ function, like urine or insulin could be measured in the liquid phase of the culture. These quality attributes are reflective of the overall function of the organ, but are indirect measures of cell health. They are also rarely measured in process steps before introduction to the bioreactor, but perhaps if they were used throughout the production process, they could help predict product quality earlier.

Given the diversity in structure and function of mature organs, there is no such thing as a typical RM tissue production process. However, whether it is a decellularization/recellularization process to generate intervertebral discs (2) or a 3D-printed scaffold seeded with iPSC-derived retinal progenitor cells (3), most *in vitro* tissue-forming processing has similar basic stages as shown in Figure 1. Cells are isolated from tissue, expanded *in vitro*, and there may or may not be manipulation such as derivation of induced pluripotent stem cells or the correction of a genetic problem. Cells are evaluated for the desired phenotype based on cell markers after this manipulation, and go into in a master cell bank (MCB) for an allogeneic treatment or patient-specific cryogenic storage. The cells are then loaded into a construct and further matured in 3D. The construct may undergo further manipulation to form the replacement organ, like folding into higher-order 4D microstructures (4) or assembly into larger organ structures.

At each of these stages, the needs of cells may change. Detecting the characteristics of cells in 3D constructs becomes difficult or impossible using the technologies that we traditionally use on dissociated cells in 2D culture. Environmental measures of cell/tissue health like glucose consumption and change in secretion profile can be monitored in bioreactors, but these are indirect. Assessing the healing organ for cellular activities like migration into functional layers and phenotypic maturation also has to change to organ-level functional assessments like urine production for kidney, and beating rhythm for heart.

Traditional manual cell culture equipment and techniques used in 2D culture are the most familiar to researchers and the least expensive, but they expose cells and tissues to non-physiologic room air (25°C/20% O_2_/0.1% CO_2_) conditions and other stresses. They introduce variability and subjective decision making into the production process. The cells experience environments which are stressful, unseen, undocumented, and largely uncontrolled.

What cytocentric technologies can we use to measure cell quality so that we not only produce, but reliably reproduce RM tissue products?

## Critical quality attributes for RM products

What defines a Critical Quality Attribute (CQA)?

A physical, chemical, biological, or microbiological property or characteristic that should be within an appropriate limit, range, or distribution to ensure the desired product quality. (5)

As per US Code of Federal Regulations (21CFR610) (6), standard criteria for product quality must be met to allow for the release of a cell therapy product. These include sterility, purity, identity, and potency. Sterility criteria are defined in 21CFR610.12, and are required to ensure viable contaminating microorganisms are not present. Identity criteria are defined in 21CFR610.14, and are critical not only for proper product labeling, but also to distinguish the product from others manufactured in the same facility. This usually means identification of cell-specific marker panels of expressed cell proteins, mRNAs, or secreted molecules. Purity criteria are defined in 21CFR610.13, and testing must be performed to ensure that the product is free of extraneous material, process residuals, or any contaminating cells. These might compromise the efficacy or safety of the final product. Potency criteria are defined in 21CFR610.3, and measure the biological activity of the product. Sterility, purity, identity, and potency are often defined as critical quality attributes (CQAs) because these product characteristics have great impact on product quality. Figure 1 shows several other important cytocentric measurements that could be CQAs, and that are often used for characterizing cell-based products and intermediates, including appearance (morphology), viability, cell count, and doubling time. In some cell products, passage number is also critical.

In the vast majority of laboratories, cell number, morphology, density, and viability are all static CQAs that are assessed through subjective visual inspection, despite clear and long-standing problems with reproducibility and reference standards (7, 8). Chemical methods used to assay cell number and viability measure membrane integrity, proliferation, and mitochondrial activity. AlamarBlue (resazurin), unlike tetrazolium salts, is a nontoxic redox indicator that has been used to assess metabolic activity over time in 2D and 3D cultures (9), However, the dye is pH, temperature, and light sensitive (10), and the uptake of AlamarBlue by cells can be altered by the surrounding construct materials (11). Intracellular ATP can be measured in organoids using assays like Cell Titre Glo and ATPlite, and the nucleic acid content of a construct can be measured with dyes like Pico Green. However, the effect of dye residues on patients present risks to their use in clinical RM products and could limit their use to sentinel organ cultures in a batch. Cytocentric technologies for in-line, continuous monitoring during cell and tissue production need to be label-free and non-destructive, as well as rigorous.

## Non-invasive quantitative imaging-based metrics of cell and population morphology in 2D cultures

The first Cytocentric Principle is to protect cell cultures from microbial contamination (1). Imaging and optical technologies may help reduce contamination risks by reducing the intrusion of physical probes. Advances in imaging and image processing increasingly transform subjective attributes into quantitative metrics that can be assessed, documented, and monitored. Moreover, non-invasive imaging and monitoring can be automated (3, 12, 13). Automation of process documentation and decision-making algorithms based on imaging parameters is not trivial. It requires rigor and process integration across several domains: image capture, image processing, image analysis, metric reporting, and data archiving. Consensus in precise and stable definitions of metrics, process and reporting standards, and systems of data capture and integration will increasingly enable process documentation and monitoring.

Following established standards (14), large field of view (LFOV) imaging strategies enable assembly of images over large areas of cell populated surfaces at pixel sizes of ∼1 micron or less. When captured with appropriate focus, processed into a seamless montage and corrected for lighting, diverse image-based tools can measure individual cells (size, area, shape, circularity) and group metrics (cell clustering, cell density, confluence, connectivity, alignment, heterogeneity). Texture metrics can assess tightly adherent cell sheets, where individual cells are not identifiable. In addition to *static* variables, *dynamic* variables (e.g., migration, proliferation, change in morphology, detachment, lysis) can also be assessed over time with quantitative imaging (Figure 1).

Phase contrast and bright-field microscopy performed with high resolution imaging, at multiple focal planes in time series, can provide extensive data sets with minimal effects on the cells. This is especially important for many cell culture experiments that require or benefit from continuous monitoring of cell growth. Image analysis software can measure changes in confluence and doubling rates, as well as track morphological changes, providing valuable insights about the health of the cells and rich data sets for additional insights. Phase contrast can be used to track morphological changes in cells following growth factor stimulation meant to enhance particular biological activities. For example, following IFNγ-treatment of MSCs, morphological subsets associated with enhanced immunosuppressive properties can be observed emerging after 24 h of IFNγ-stimulation (15).

Quantitative imaging also can be used effectively for quality/reproducibility control for cell sourcing. Performance based clone selection, i.e., starting a fabrication process by selecting individual cell clones based on defined quantitative metrics will reduce variability in outcome. Quantitative imaging metrics can also feed machine learning algorithms that define CQAs for starting materials and clones that are linked to critical downstream product CQAs.

Digital Holographic Microscopy (DHM) is the most common form of quantitative phase imaging and allows for non-invasive imaging of living cells over time. DHM allows for real-time detection and quantification of both single cells and cell populations without the need to disrupt routine cell culture. DHM has been used on a wide range of cells, ranging from bacteria and protozoa to mammalian cells (16). In addition, DHM can be used to study a range of dynamic cellular parameters from movement, proliferation, and cell death to morphological, phenotypical, and behavioral changes (17). Moreover, as DHM is non-invasive and introduces minimal external stress to the cells, the technology is suitable for long term live-cell imaging. This is especially important for cells that are difficult to obtain or grow. DHM is a uniquely cytocentric measurement.

## Determining CQAs in 3D cultures

Over the past decade, a broad spectrum of 3D culture models employing scaffolds, spheroids, organoids, organs-on-chips (OOC), and bioprinted scaffolds have emerged to better recapitulate the complexity of *in vivo* tissues. In contrast to 2D traditional cell culture, these models may be more physiologically relevant *in vitro* systems for drug development and screening, disease modeling, regenerative medicine, and fundamental biomedical research (18–22).

OOC technologies enable the precise control of the micro-environmental factors that can modulate cell conditions, 3D tissue development, and tissue responses to stimuli. This allows OOC systems to provide more *in vivo*-like environmental, biological, biomechanical, and biochemical cues to the cells for creating progressively more complex tissue and organ models (23). Co-culture, 3D tissue-tissue interfaces, cyclic shear, stretch, electrical biomechanical signals, and controlled oxygen microenvironments all provide cells in culture a more biologically relevant environment (19, 24, 25).

Microfluidic devices also provide a platform where the cell and tissue culture conditions and metabolic activities can be monitored non-destructively and continuously. This capability of the OOC technology is still in its infancy, but has tremendous potential for regenerative medicine and tissue engineering applications (26, 27). Miniaturized electrodes integrated into the microfluidic devices have been used for obtaining spatiotemporal information on cell attachment, growth, morphology, function, differentiation, transepithelial-endothelial electrical resistance (TEER), electrophysiological function, biomechanical contractility, and motility in microfluidic and OOC devices (26–29). Many of these measurements could be important early indicators of product quality and could become CQAs for relevant cell manufacturing processes.

This illustrates the need for more development of technologies such as sensors and integrated electrodes that can collect and communicate cell information to the culture controlling system automatically for documentation, analysis, and adjustments in environment for higher order tissue structures.

The perfusing media in OOC models can simultaneously be sampled to monitor critical process parameters (CPP) in the cell environment that also not only reflect culture health, but also control it. Allowing the cells to grow undisturbed in optimal, reproducible conditions is fundamental to cytocentric measurements.

## Critical process parameters as controllable measurements of cell health for RM

A Critical Process Parameter (CPP) is defined as a process parameter whose variability has an impact on a critical quality attribute and therefore should be monitored or controlled to ensure the process produces the desired quality (5). The use of non-destructive, non-intrusive sensors is in accord with cytocentric manufacturing approaches. Electrochemical sensors can measure glucose, lactate, L-glutamate and other analytes that can indicate cell culture conditions (Figure 1) (30–33). Optical sensors employ a range of analyte sensitive indicator dyes that emit a signal upon illumination and interaction with the target analyte (34, 35). Luminescence optical sensors have most successfully been used in sensing oxygen in microfluidic devices (19, 26, 30, 34). Temperature and pH have also been measured in microfluidic devices using optical sensors (27, 35). However, they have been integrated into OOC devices only in a limited way.

In-line process analytics of cytocentric CPPs such as O_2_, CO_2_, lactate, temperature, cell density, viability and cell morphometrics are key to ensuring the quality of growing cell and tissue cultures. Dissolved oxygen (DO) and pH are two critical parameters that are known to affect cell and tissue metabolism, morphology, gene expression, and cell health, and protein production (36). For analysis of 3D cultures during production, some promising innovations employ the functionalization of ECM materials with luminescent optical sensor nanoparticles for live oxygen monitoring (37), as well as the use of dielectric impedance spectroscopy to characterize cell viability (38).

As oxygen does not dissolve easily in aqueous media, local DO around the cell is a function of local cell density, metabolic rate, fluid convection and the path of O_2_ diffusion. Studies have incorporated electrochemical sensors to monitor oxygen and lactate in liver spheroids cultured in 96 well plates (39). These technologies may be able to more finely control tissue microenvironments for the development of physiologically relevant healthy and diseased tissue models.

However, monitoring of DO and pH does not often begin until the cells enter the bioreactor as constructs. Cell culture incubators are usually at 5% CO_2_ and 18%–20% O_2_. The CO_2_, in conjunction with a carbonate buffer system in many cell culture media, will stabilize the pericellular pH between 7.2–7.4. If the medium contains phenol red, researchers open the incubator and take cultures out to visually estimate the color of the media for pH. This is a highly subjective and irreproducible assessment.

There are few readily available sensing technologies made specifically for measuring DO and pH levels in small 2D culture vessels. Fill volumes are far too low to cover these sensors. Optical sensing technologies, which consist of fluorescent sensor stickers and a separate reading device, have the potential to provide DO and pH monitoring in small 2D culture vessels because they can be smaller. However, outfitting each cell culture vessel with optical sensors currently proves cumbersome in terms of price, incubator space, and workflow. Most researchers forgo monitoring of DO and pH altogether in the 2D phase of RM processes. However, without these fundamental CPPs, it may be impossible to insure sufficient control of the cellular environment to produce a consistent RM product.

To achieve truly manufacturable RM products, environmental control, modeling, or sensing technologies must be implemented from the very beginning of cell and tissue culture processes (Figure 1). In a perfect scenario, *ex vivo* cells and tissues would have a perfectly simulated *in vivo* experience. Ideally, every important analyte, metabolite, and variable should be understood, recorded, and controlled through a broad array of sensors and automatic-control feedback loops. Cytocentric measurements would be universally performed in all of the diverse vessel types that cells encounter in every step throughout the tissue production process. Presently, this is not realistic, but it is time to lay the foundation and take the first steps. The National Institute of Standards and Technologies is active in the area of establishing new standards for cell measurements (link).

Potential CQAs and CPPs may vary between cell sources, tissue types, and product applications. For example, a metabolic shift may signal tissue maturation in liver, but not in skin. Defining the optimal conditions for different RM products will require knowledge and control of process parameters and their direct association with outcome metrics.

As we develop this knowledge, the ultimate goal is to use these data for active, real-time in-process adaptation. Detection of a product deviation, identified using imaging, can enable flagging of product variation and a diversity of in-process intervention strategies, such as oxygen tension modulation, targeted picking, or thinning routines (13). Cytocentric analytics and Smart Manufacturing algorithms are needed to identify CQAs and CPPs that reliably result in reproducible RM tissue products.

## Conclusions

Cytocentric measurements are improving, particularly with development of imaging-based metrics. However, we need to continue the evolution from manual, static measurements to automated, dynamic measurements that allow for real-time measurement and control of process parameters. Technical challenges remain in getting the information we need about the constantly changing tissue cultures in our care. The use of continuous measurement technologies like the electrochemical and optical sensors in bioreactors, microfluidic devices, and tissue culture systems need to be more widespread. The knowledge obtained from such sophisticated *in vitro* systems is essential to developing functional and truly reproducible *in vivo*-like tissues for Regenerative Medicine.

## Data Availability

The original contributions presented in the study are included in the article/Supplementary Material, further inquiries can be directed to the corresponding author.
